# Towards a comprehensive structural variation map of an individual human genome

**DOI:** 10.1186/gb-2010-11-5-r52

**Published:** 2010-05-19

**Authors:** Andy W Pang, Jeffrey R MacDonald, Dalila Pinto, John Wei, Muhammad A Rafiq, Donald F Conrad, Hansoo Park, Matthew E Hurles, Charles Lee, J Craig Venter, Ewen F Kirkness, Samuel Levy, Lars Feuk, Stephen W Scherer

**Affiliations:** 1 Department of Molecular Genetics, University of Toronto, 1 King's College Circle, Toronto, Ontario M5S 1A8, Canada; 2 The Centre for Applied Genomics, The Hospital for Sick Children, 101 College Street, Toronto, Ontario M5G 1L7, Canada; 3 Wellcome Trust Sanger Institute, The Wellcome Trust Genome Campus, Hinxton, Cambridge CB10 1SA, UK; 4 Department of Pathology, Brigham and Women's Hospital and Harvard Medical School, 221 Longwood Avenue, Boston, Massachusetts 02115, USA; 5 J Craig Venter Institute, 9740 Medical Center Drive, Rockville, Maryland 20850, USA; 6 Department of Genetics and Pathology, Rudbeck Laboratory, Uppsala University, Uppsala 75185, Sweden

## Abstract

**Background:**

Several genomes have now been sequenced, with millions of genetic variants annotated. While significant progress has been made in mapping single nucleotide polymorphisms (SNPs) and small (<10 bp) insertion/deletions (indels), the annotation of larger structural variants has been less comprehensive. It is still unclear to what extent a typical genome differs from the reference assembly, and the analysis of the genomes sequenced to date have shown varying results for copy number variation (CNV) and inversions.

**Results:**

We have combined computational re-analysis of existing whole genome sequence data with novel microarray-based analysis, and detect 12,178 structural variants covering 40.6 Mb that were not reported in the initial sequencing of the first published personal genome. We estimate a total non-SNP variation content of 48.8 Mb in a single genome. Our results indicate that this genome differs from the consensus reference sequence by approximately 1.2% when considering indels/CNVs, 0.1% by SNPs and approximately 0.3% by inversions. The structural variants impact 4,867 genes, and >24% of structural variants would not be imputed by SNP-association.

**Conclusions:**

Our results indicate that a large number of structural variants have been unreported in the individual genomes published to date. This significant extent and complexity of structural variants, as well as the growing recognition of their medical relevance, necessitate they be actively studied in health-related analyses of personal genomes. The new catalogue of structural variants generated for this genome provides a crucial resource for future comparison studies.

## Abstract 2

A comprehensive map of structural variation in the human genome provides a reference dataset for analyses of future personal genomes.

## Background

Comprehensive catalogues of genetic variation are crucial for genotype and phenotype correlation studies [1-8], in particular when rare or multiple genetic variants underlie traits or disease susceptibility [9,10]. Since 2007, several personal genomes have been sequenced, capturing different extents of their genetic variation content (Additional file 1) [1-8,11]. In the first publication (J Craig Venter's DNA named HuRef) [1], variants were identified based on a comparison of the Venter assembly to the National Center for Biotechnology Information (NCBI) reference genome (build 36). In total, 3,213,401 SNPs and 796,167 structural variants (SVs; here SV encompasses all non-SNP variation) were identified in that study. Similar numbers of SNPs, but significantly less SVs (ranging from approximately 137,000 to approximately 400,000) are reported in other individual genome sequencing projects [2-4,6-8,11]. It is clear that even with deep sequence coverage, annotation of structural variation remains very challenging, and the full extent of SV in the human genome is still unknown.

Microarrays [12-14] and sequencing [15-18] have revealed that SV contributes significantly to the complement of human variation, often having unique population [19] and disease [20] characteristics. Despite this, there is limited overlap in independent studies of the same DNA source [21,22], indicating that each platform detects only a fraction of the existing variation, and that many SVs remain to be found. In a recent study using high-resolution comparative genomic hybridization arrays, the authors found that approximately 0.7% of the genome was variable in copy number in each hybridization of two samples [19]. Yet, these experiments were limited to the detection of unbalanced variation larger than 500 bp, and the total amount of variation between two genomes would therefore be expected to exceed 0.7%.

**Figure 1:**
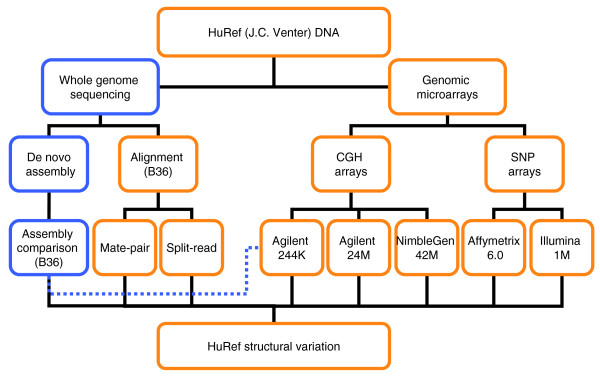
**Overall workflow of the current study**. Two distinct technologies were used to identify SV in the Venter genome: whole genome sequencing and genomic microarrays. The sequencing experiments, the construction of the Venter genome assembly, and the assembly comparison with NCBI build 36 (B36) reference had been completed in previous studies [1,16,39]. Hence, these experiments are shown as blue boxes. The scope of the current study is denoted in orange boxes. We re-analyzed the initial sequencing data, and searched for SVs in sequence alignments by the mate-pair and split-read approaches. We also used three distinct comparative genomic hybridization (CGH) array platforms: Agilent 24 M, NimbleGen 42 M and Agilent 244 K. Unlike the other array platforms, which were designed based on the B36 assembly, the Agilent 244 K targeted scaffold segments unique to the Celera/Venter assembly. To denote this, Figure 1 shows a dotted line connecting between the assembly comparison outcome and the Agilent 244 K box. Finally, the Affymetrix 6.0 and Illumina 1 M SNP arrays were also used in the present study.

**Figure 2:**
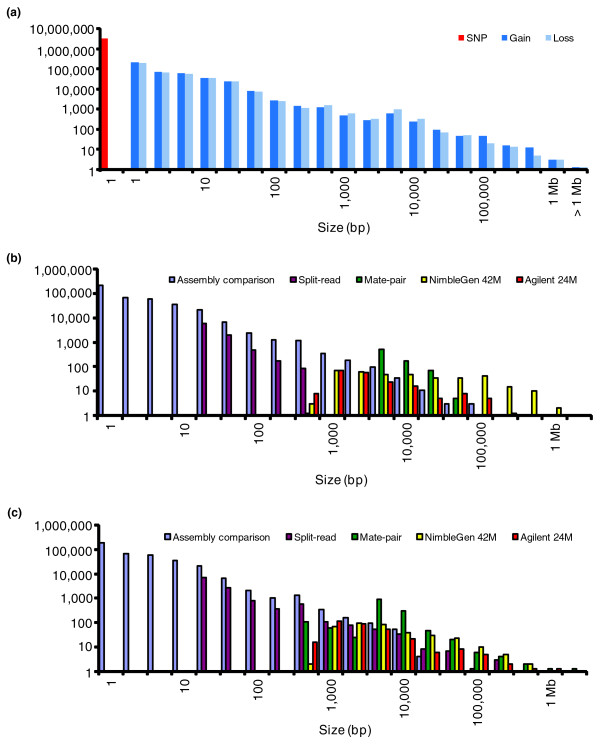
**Size distribution of genetic variants**. **(a) **A non-redundant size spectrum of SNP and CNV (including indels) and a breakdown of the proportion of gain to loss. The indel/CNV dataset consists of variants detected by assembly comparison, mate-pair, split-read, NimbleGen 42 M comparative genomic hybridization (CGH) and Agilent 24 M. The results show that the number and the size of variants are negatively correlated. Although the proportions of gains and losses are quite equal across the size spectrum, there are some deviations. Losses are more abundant in the 1 to 10 kb range, and this is mainly due to the inability of the 2-kb and 10-kb library mate-pair clones to detect insertions larger than their clone size. The opposite is seen for large events, where duplications are more common than deletions, which may be due to both biological and methodological biases. The increase in the number of events near 300 bp and 6 kb can be explained by short interspersed nuclear element (SINE) and long interspersed nuclear element (LINE) indels, respectively. The general peak around 10 kb corresponds to the interval with the highest clone coverage. **(b) **Size distribution of gains (insertions and duplications) highlighting the detection range of each methodology. The split-read method is designed to capture insertions from 11 bp to the size of a Sanger-based sequence read (approximately 1 kb). There is no insertion detected in the size range between the 2 kb and 10 kb library using the mate-pair approach. Furthermore, due to technical limitations, large gains (≥ 100,000 bp) cannot be identified with the sequencing-based approaches, while these are readily identified by microarrays. **(c) **Size distribution of deletions.

Our objective in the present study was to annotate the full spectrum of genetic variation in a single genome. We used the previously sequenced Venter genome due to the availability of DNA and full access to genome sequence data. The assembly comparison method presented in the initial sequencing of this genome [1] discovered an unprecedented number of SVs in a single genome; however, the approach relied on an adequate diploid assembly. As there are known limitations in assembling alternative alleles for SV [1], we expected that there was still a significant amount of variation to be found. In an attempt to capture the full spectrum of variation in a human genome, this current study uses multiple sequencing- and microarray-based strategies to complement the results of the assembly comparison approach in the Levy *et al. *[1] study. First, we detect genetic variation from the original Sanger sequence reads by direct alignment to NCBI build 36 assembly, bypassing the assembly step. Furthermore, using custom high density microarrays, we probe the Venter genome to identify variants in regions where sequencing-based approaches may have difficulties (Figure 1). We discover thousands of new SVs, but also find biases in each method's ability to detect variants. Our collective data reveal a continuous size distribution of genetic variants (Figure 2a) with approximately 1.58% of the Venter haploid genome encompassed by SVs (39,520,431 bp or 1.28% as unbalanced SVs and 9,257,035 bp or 0.30% as inversions) and 0.1% as SNPs (Table 1, Figure 2). While there is still room for improvement, our results give the best estimate to date of the variation content in a human genome, provide an important resource of SVs for other personal genome studies, and highlight the importance of using multiple strategies for SV discovery.

**Table 1:** Structural variants detected by different methods

Method	Type	Number	Minimum size (bp)	Median size (bp)	Maximum size (bp)	Total size (bp)
*Assembly comparison*^a^	*Homo. insertion*	*275,512*	*1*	*2*	*82,711*	*3,117,039*
	*Homo. deletion*	*283,961*	*1*	*2*	*18,484*	*2,820,823*
	*Hetero. insertion*	*136,792*	*1*	*1*	*321*	*336,374*
	*Hetero. deletion*	*99,814*	*1*	*1*	*349*	*250,300*
	*Inversion*	*88*	*102*	*1,602*	*686,721*	*1,627,871*
Mate-pair	Insertion	780	346	3,588	28,344	3,880,544
	Deletion	1,494	340	3,611	1,669,696	10,531,345
	Inversion	105	368	3,121	2,026,495	8,068,541
Split-read	Insertion	8,511	11	16	414	224,022
	Deletion	11,659	11	18	111,714	1,764,522
Agilent 24 M	Duplication	194	445	1,274	113,465	1,065,617
	Deletion	319	439	1,198	852,404	2,779,880
NimbleGen 42 M	Duplication	366	448	4,665	836,362	11,292,451
	Deletion	358	459	2,460	359,736	3,861,282
Affymetrix 6.0	Duplication	17	8,638	42,798	640,474	2,011,557
	Deletion	21	2,280	13,145	856,671	1,978,028
Illumina 1 M	Duplication	3	11,539	22,148	87,670	121,357
	Deletion	9	8,576	32,199	145,662	431,131
Custom Agilent 244 k	Duplication	44	219	1,356	8,737	98,529
	Deletion	7	170	332	2,258	4,130
**Non-redundant total^b^**	**Insertion/duplication**	**417,206**	**1**	**1**	**836,362**	**19,981,062**
	**Deletion**	**390,973**	**1**	**2**	**1,669,696**	**19,539,369**
	**Inversion**	**167**	**102**	**1,249**	**2,026,495**	**9,257,035**

^a^We used an italicized font to distinguish the results from the Levy *et al. *[1] study. Moreover, from that previous study, we included all homozygous indels, heterozygous indels, indels embedded within simple, bi-allelic, and non-ambiguously mapped heterozygous mixed sequence variants, and only those inversions whose size is at most 3 Mb. ^b^Complete data are presented in Additional files 19, 20 and 21. Non-redundant variation size distribution is presented in Figure 2a.

## Results

Several different analytical and experimental strategies were employed to exhaustively analyze the Venter genome for SV. An overview of the different analyses performed is shown in Figure 1.

### Sequencing-based variation

We first used computational strategies to extract additional SV information from the existing Sanger-based sequencing data generated as paired-end (or mate-pair) reads from clone libraries of defined size [1]. First, we adopted a paired-end mapping approach [15,17,18] and aligned 11,346,790 mate-pairs from libraries with expected clone sizes of 2, 10 or 37 kb (Additional file 2) to the NCBI build 36 assembly. We found that 97.3% of mate-pairs had the expected mapping distance and orientation. Mate-pairs discordant in orientation or mapping distance were used to identify variants, and we required each event to be supported by at least two clones. In total, this strategy was used to identify 780 insertions, 1,494 deletions and 105 inversions (Figure 1; Table 1; Additional file 3). In an independent analysis of the same underlying sequencing data, we then captured SVs by examining the alignment profiles of 31,546,016 paired and unpaired reads to search for intra-alignment gaps [23]. The presence of an intra-alignment gap in the sequence read (query sequence) or in the reference genome (target sequence) would indicate a putative insertion or deletion event, respectively. The identification of such a 'split-read' alignment signature complements the mate-pair approach, as significantly smaller insertions and deletions can be discovered. We required at least two overlapping split-reads having an alignment gap >10 bp to call a variant. A total of 8,511 insertions and 11,659 deletions ranging from 11 to 111,714 bp in size were identified (Figure 1; Table 1; Additional file 4).

### Array based variation

We used two ultra-high density custom comparative genomic hybridization (CGH) array sets and two commonly used SNP genotyping arrays to identify relative gains and losses. A significant amount of variation was detected from the two custom CGH arrays: an Agilent oligonucleotide array set with 24 million features (Agilent 24 M) [7], and a NimbleGen oligonucleotide array set containing 42 million features (NimbleGen 42 M) [19]. The Agilent platform identified 194 duplications and 319 deletions, while the NimbleGen array set detected 366 gains and 358 losses, ranging in size from 439 bp to 852 kb, in Venter (Figure 1; Table 1; Additional files 5 and 6). Furthermore, we scanned the Venter genome using Affymetrix SNP Array 6.0 and Illumina BeadChip 1 M, and the results are summarized in Table 1 plus Additional files 7 and 8.

Most microarrays used for CNV analyses are designed based on the NCBI assemblies. Therefore, any region where the reference exhibits the deletion allele of an indel, or sequences mapping to gaps in the assembly, will not be targeted. In previous studies [16,24], many unknown DNA segments were identified to have no or poor alignment to the NCBI reference when compared to the Celera R27C assembly. To capture genetic variation in such potentially novel sequences, we designed a custom Agilent 244 K array to target those scaffold sequences at least 500 bp in length. We then performed CGH on seven HapMap individuals and detected 231 regions (101 gains and 130 losses) in 161 scaffolds to be variable (Additional file 9). Of these, we found 44 gains and 7 losses in 36 Celera scaffolds were specific to Venter (Figure 1, Table 1). Using paired-end mapping, as well as cross-species genome comparison with the chimpanzee, we were able to find a placement in NCBI build 36 for 25 of 36 scaffolds that were copy number variable in Venter. Two of the scaffolds were mapped to regions containing assembly gaps, 15 of 25 anchored scaffolds corresponded to insertion events also detected elsewhere [15,18], and the remaining eight represent new insertion findings (Additional file 10).

### Validation of findings

We used several computational and experimental approaches to validate our SV findings. We performed experimental validation by PCR amplification and gel-sizing and confirmed 89 of the 96 (93%) SVs predicted by sequence analysis (Additional files 11 and 12). Using quantitative real-time PCR (qPCR), we validated 20 of 25 (80.0000%) CNVs detected by microarrays, and most of these CNVs were from the custom Agilent 244 K array covering sequences not in the NCBI assembly (Additional file 13). Inversion predictions were tested by fluorescence *in situ *hybridization (FISH) [25]. In one such finding, a predicted 1.1-Mb inversion at 16p12 was identified to be homozygous in Venter and in all of the seven additional HapMap samples from four populations tested, suggesting that the reference at this locus represents a rare allele, or is incorrectly assembled (Additional file 14).

We then compared the SVs identified here with the previous assembly comparison-based analysis of the same genome [1], and found that 11,140 variants were in common. We noticed that our multi-platform method excelled in calling large variants. In fact, even after excluding all of the small variants (≤ 10 bp) from the previous Levy *at al*. study [1], we still observed that the current study tended to find larger SVs (a current average of 1,909.3 bp now versus a previous average of 113.4 bp). Additional file 15 shows that the sensitivity of assembly comparison dropped as size increased to over 1 kb, and the proportion of larger SVs significantly increased as a result of the present study (Figure 2b, c).

Finally, we determined the number of calls in this study that were either verified by another platform in this study or found in the Database of Genomic Variants [12]. In total, we computationally confirmed 15,642 (65.6%) of our current calls: 6,301 were gains; 9,726 were losses; and 65 were inversions.

### Cross-platform comparison

**Figure 3:**
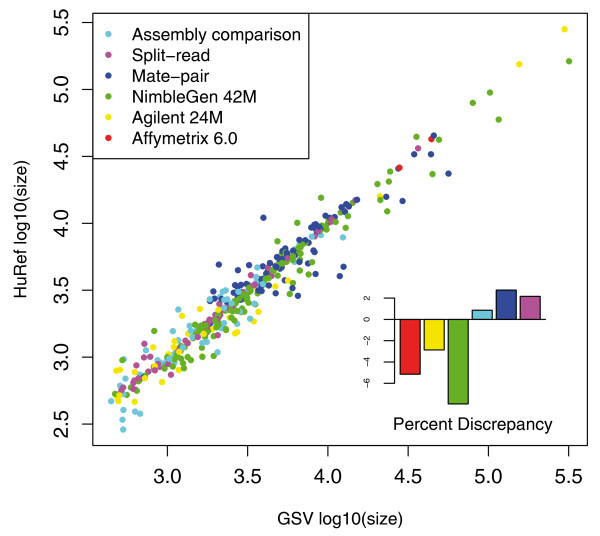
**Agreement between the non-redundant set of Venter CNVs and genotype-validated variable loci**. The agreement between sites identified by different detection methods was measured by the percentage of reciprocal overlap between the estimated size for the non-redundant set of Venter variants and the estimated size for the CNVs generated and genotyped in the Genome Structural Variation (GSV) population genetics study [19]. Two sites were considered overlapping if the reciprocal overlap among their estimated sizes was ≥ 50%. The lower right corner plot summarizes the mean discrepancy between Venter and GSV loci sizes, as a proportion of the GSV-estimated CNV size.

We performed an in-depth analysis of the characteristics of the variants detected by each of the methods. First, by contrasting against a population-based study [19], we observed highly similar size estimates for the same underlying SVs between methods (Figure 3). With sufficient genome coverage of clones with accurate and tight insert size, the mate-pair method yields precise variation size. Similarly, the split-read approach gives nucleotide resolution breakpoints, while the high-density CGH and SNP arrays have dense probe coverage to accurately identify the start and end points of SVs. Overall, our multiple approaches are highly robust in estimating variant size.

Next, we compared the variants discovered by the two whole genome CGH array sets, NimbleGen 42 M and Agilent 24 M, and investigated the primary reason for the discordance between the two data sets. Not surprisingly, a substantial portion of the discordant calls can be explained by the difference in probe coverage. In fact, approximately 70% of the unique calls on the NimbleGen 42 M array had inadequate probe coverage on the Agilent 24 M array to be able to call variants, and approximately 30% *vice versa *(Additional file 16). After that, we compared the number of calls uniquely identified by the SNP-genotyping microarrays, and we identified 12 and 0 novel SVs contributed by Affymetrix 6.0 and Illumina 1 M, respectively. Of the 12 new Affymetrix calls, 9 are located in complex regions containing blocks of segmental duplications.

Subsequently, when looking for enrichment of genomic features among variants detected by different approaches, we found that there was a significant enrichment (*P *< 0.01) of short interspersed nuclear elements (SINEs) in deletions called by sequencing-based approaches (mate-pair and split-read), but not in deletions called by the microarrays. Microarrays have low sensitivity for detecting copy number change of SINEs (for example, Alu elements), as these regions cannot be uniquely targeted by short oligo probes, and over-saturation of probe fluorescence would prevent an accurate high copy count. Meanwhile, the sequencing methods employed here do not rely on alignments within the repeat itself, and consequently they are readily able to detect gains and losses of these high-copy repeats. The complete result for enrichment of SVs with various genomic features is shown in Additional file 17.

Finally, one of the main challenges of genome assembly is to correctly assemble both alleles in regions of SV. To identify heterozygous events among the split-read indels, we searched for evidence of an alternative allele. Indels were determined to be heterozygous if two or more sequence reads could be aligned that supported the NCBI build 36 allele. From the split-read dataset alone, we identified 4,476 of 8,511 (52.6%) insertions and 6,906 of 11,659 (59.2%) deletions as heterozygous. Additionally, we found that of the 10,834 split-read indels that overlapped with results from the Levy *et al. *study [1], 4,332 events annotated as heterozygous in our results were previously classified as homozygous (Additional file 4). These differences highlight the difficulty of assembling both alternative alleles in regions of SV, leading to an underestimate of the heterozygosity in Levy *et al. *[1].

### The total variation content of the Venter genome

In an attempt to estimate the total variation content in the Venter genome, we combined the SVs previously described in the Venter genome in the Levy *et al. *paper [1] with the variants discovered in this study, to generate a non-redundant set of variants. We determined that 48,777,466 bp was structurally variable, of which 19,981,062 bp belonged to gains, 19,539,369 bp to losses, and 9,257,035 bp to balanced inversions (Table 1). A vast majority of this variation was discovered in the current analyses (83.3% or 40,625,059 bp) of the Venter genome. Therefore, our significant contribution in detecting novel calls underscores the importance of using multiple analysis strategies for detecting SV in the human genome. See Additional file 18 for the location of SVs >1 kb, and Additional files 19, 20 and 21 for a complete list of variation in the Venter genome.

### Comparison with other personal genomes

**Figure 4:**
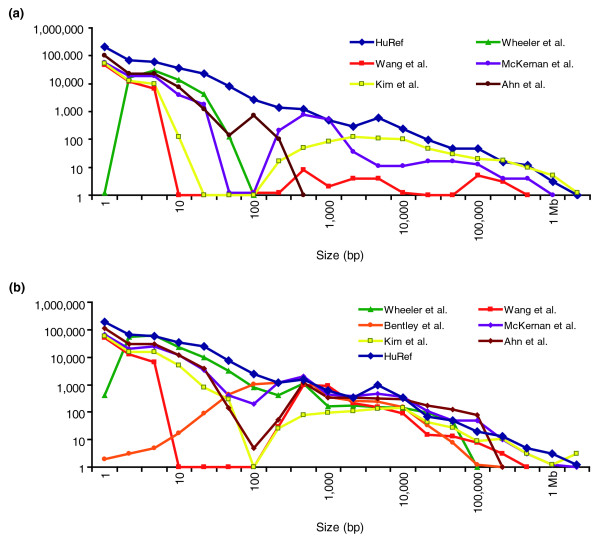
**Difference in the size distributions of reported indels/CNVs in published personal genome sequencing studies**. The graphs show variation found in a few personal genome sequencing studies [1-4,6-8]. These diagrams indicate that multiple approaches are needed for better detection of CNVs. Here, the total variant set in the Venter genome found in both the Levy *et al. *[1] and the current study is displayed. Unlike the current study where the size of mate-pair indels is equal to the difference between the mapping distance and the expected insert size, the SVs in the Ahn *et al. *[6] study are only based on the mapping distance. Besides the NGS data, we have also included the variants detected by the high density Agilent 24 M data in the Kim *et al. *[7] study. In Wheeler *et al. *[2], insertions identified by intra-read alignment would be limited by the size of the sequencing read; hence, large insertions beyond the read length were not detected. Wang *et al. *[4], Kim *et al.*, and McKernan *et al. *[8] detected small variants based on split-reads and large ones based on mate-pairs and microarrays, but failed to detect variation between these size ranges. Also, see Additional file 1. **(a) **Insertion and duplication size distribution. **(b) **Deletion size distribution.

When we compared the complete set of Venter's SVs with those from other published genomes [2-4,6-8] (Additional file 1), we found that 209,493/808,345 (25.9%) of the Venter variants overlapped variants described in one or more of the other six studies. Upon examining the size distribution of variants from different studies, particularly the size of insertions and duplications, we realized that studies based primarily on next generation sequencing (NGS) data for variation calling were unable to identify calls in certain size ranges (Figure 4). These results further signify that, at present, multiple approaches are needed to capture SVs across the entire size spectrum. The most obvious limitation is that short next generation sequencing NGS reads/inserts fail to capture insertion events greater than the size of the reads/inserts.

### Functional importance of structural variation

Next, we analyzed the complete set of SVs in Venter for overlap with features of the genome with known functional significance, which might influence health outcomes (Table 2). We found 189 genes to be completely encompassed by gains or losses, 4,867 non-redundant genes (3,126 impacted by gains and 3,025 by losses) whose exons were impacted, and 573 of these to be in the Online Mendelian Inheritance in Man (OMIM) Disease database (Additional files 22, 23, 24, 25 and 26). However, there was an overall paucity of SV (*P *≥ 0.999) overlapping exonic sequences of genes associated with autosomal dominant/recessive diseases, cancer disease, and imprinted and dosage-sensitive genes. In general, there is an absence of variation in both exonic and regulatory sequences, such as enhancers, promoters and CpG islands, in the genome of this individual.

**Table 2:** Genomic landscape and structural variants in the Venter genome*

	Total non-redundant gains^b^			Total non-redundant losses^c^		
		
Genomic feature (number of entries)^a^	Number of (%) genomic features	Number of (%) structural variants	*P*-values	Number of (%) genomic features	Number of (%) structural variants	*P*-values
RefSeq gene loci^d ^(20,174)	14,268 (70.72%)	159,250 (38.17%)	0.000	13,951 (69.15%)	149,568 (38.26%)	0.000
RefSeq gene entire transcript loci^e ^(20,174)	101 (0.50%)	41 (0.01%)	0.000	91 (0.45%)	47 (0.01%)	0.000
RefSeq gene exons^f ^(20,174)	3,126 (15.50%)	3,890 (0.93%)	0.999	3,025 (14.99%)	3,723 (0.95%)	0.999
Enhancer elements (837)	80 (9.56%)	85 (0.02%)	0.999	84 (10.04%)	93 (0.02%)	0.999
Promoters (20,174)	2,007 (9.95%)	2,071 (0.50%)	0.999	1,812 (8.98%)	1,922 (0.49%)	0.999
Stop codons^g ^(30,885)	225 (0.73%)	99 (0.02%)	0.000	272 (0.88%)	134 (0.03%)	0.563
OMIM disease gene loci (3,737)	1,658 (44.37%)	20,589 (4.93%)	0.000	1,664 (44.53%)	19,396 (4.96%)	0.000
OMIM disease gene exons (3,737)	367 (9.82%)	458 (0.11%)	0.999	383 (10.25%)	492 (0.13%)	0.999
Autosomal dominant gene loci (316)	247 (78.16%)	2,773 (0.66%)	0.023	245 (77.53%)	2,593 (0.66%)	0.031
Autosomal dominant gene exons (316)	60 (18.99%)	70 (0.02%)	0.999	64 (20.25%)	78 (0.02%)	0.999
Autosomal recessive gene loci (472)	386 (81.78%)	3,931 (0.94%)	0.065	402 (85.17%)	3,749 (0.96%)	0.009
Autosomal recessive gene exons (472)	58 (12.29%)	78 (0.02%)	0.999	86 (18.22%)	109 (0.03%)	0.999
Cancer disease gene loci (363)	301 (82.92%)	4,202 (1.01%)	0.651	307 (84.57%)	3,899 (1.00%)	0.821
Cancer disease gene exons (363)	66 (18.18%)	85 (0.02%)	0.999	71 (19.56%)	98 (0.03%)	0.999
Dosage sensitive gene loci (145)	120 (82.76%)	2,995 (0.72%)	0.604	125 (86.21%)	2,794 (0.71%)	0.728
Dosage sensitive gene exons (145)	39 (26.90%)	51 (0.01%)	0.999	41 (28.28%)	58 (0.01%)	0.999
Genomic disorders (52)	50 (96.15%)	14,178 (3.40%)	0.999	51 (98.08%)	13,373 (3.42%)	0.996
Pharmacogenetic gene loci (186)	97 (52.15%)	853 (0.20%)	0.517	96 (51.61%)	838 (0.21%)	0.105
Pharmacogenetic gene exons (186)	21 (11.29%)	27 (0.01%)	0.998	23 (12.37%)	29 (0.01%)	0.984
Imprinted gene loci (59)	39 (66.10%)	405 (0.10%)	0.989	37 (62.71%)	378 (0.10%)	0.982
Imprinted gene exons (59)	13 (22.03%)	15 (0.00%)	0.998	11 (18.64%)	13 (0.00%)	0.999
MicroRNAs (685)	8 (1.17%)	9 (0.00%)	0.785	11 (1.61%)	9 (0.00%)	0.836
GWAS loci (419)	415 (99.05%)	9,413 (2.26%)	0.000	416 (99.28%)	8,852 (2.26%)	0.000
GWAS SNPs (419)	1 (0.24%)	1 (0.00%)	0.786	2 (0.48%)	2 (0.00%)	0.810
CpG islands (14,867)	287 (1.93%)	1,516 (0.36%)	0.999	299 (2.01%)	1,508 (0.39%)	0.999
DNAseI hypersensitivity sites (95,709)	6,524 (6.82%)	7,165 (1.72%)	0.999	6,392 (6.68%)	6,914 (1.77%)	0.999
Recombination hotspots (32,996)	16,839 (51.03%)	30,315 (7.27%)	0.000	16,211 (49.13%)	28,407 (7.27%)	0.000
Segmental duplications (51,809)	17,172 (33.14%)	13,864 (3.32%)	0.999	16,518 (31.88%)	13,177 (3.37%)	0.999
Ultra-conserved elements (481)	2 (0.42%)	2 (0.00%)	0.999	2 (0.42%)	2 (0.00%)	0.999
Affy 6.0 SNPs^h ^(907,691)	1,556 (0.17%)	389 (0.09%)	0.999	3,022 (0.33%)	934 (0.24%)	0.999
Illumina 1 M SNPs^i ^(1,048,762)	2,318 (0.22%)	601 (0.14%)	0.999	4,789 (0.46%)	1,536 (0.39%)	0.999

*This table shows how structural variation affects different functional annotations and sequence characteristics in the Venter genome. The leftmost column shows the names and total number of genomic features. The rest of the table is divided between gains and losses. Within the gain category, the first left column shows the number of (and percentage of total) genomic features impacted, and the second column shows the corresponding number of (and percentage of total) gain variants, and the last column shows the significance of the overlap as determined by simulations. An identical format is used for the losses. ^a^See Additional file 17 for a list of data sources. ^b^Based on a non-redundant list of 417,206 gains and insertions detected in this and the Levy *et al. *[1] study of the Venter genome. ^c^Based on a non-redundant list of 390,973 deletions detected in this and the Levy *et al. *[1] study of the Venter genome. ^d^Genes where a structural variant resides anywhere within the transcript (exonic and intronic). ^e^Genes from the RefSeq data set where the entire transcript locus is encompassed by the structural variant. ^f^Genes from the RefSeq data set where exonic sequence is impacted by the structural variant. The non-redundant number of genes altered in some way by duplications and deletions is 4,867. ^g^Structural variants that overlap/impact a stop codon from the RefSeq gene set. ^h^Probes on the Affymetrix 6.0 Commercial array. ^i^Probes on the Illumina 1 M array. GWAS, genome-wide association studies; OMIM, Online Mendelian Inheritance in Man.

Currently, direct-to-consumer testing companies and genome-wide association studies mainly use microarray-based SNP data [26,27], but SVs are typically not considered. Venter indels/CNVs, however, overlap with 4,565 and 7,047 of SNPs on the Affymetrix SNP-Array 6.0 and Illumina-BeadChip 1 M products (two commonly used arrays) potentially impacting genotype calling, most notably when deletions are involved.

**Figure 5:**
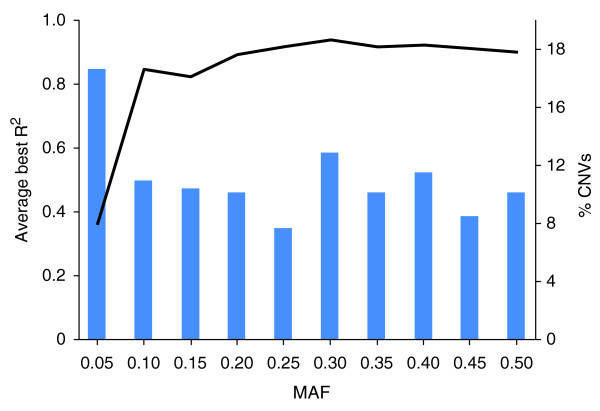
**Tagging pattern for HuRef SVs as a function of its minimum allele frequency (MAF)**. Linkage disequilibrium is depicted as the best *r*^2 ^between a SV and a HapMap SNP in 120 Europeans (CEU). There were a total of 405 bi-allelic polymorphic SV sites of overlap between GSV and HuRef loci; 24% of the SV loci have a HapMap SNP with *r*^2 ^< 0.8 in CEU, a cutoff below which HuRef CNVs would not be imputed simply by SNP detection. The line graph corresponds to the left y-axis, while the bar graph corresponds to the right y-axis. It should be noted that this analysis is performed on a small subset of bi-allelic SVs and that the ability to impute a larger fraction of SVs based on common SNPs would be even lower.

Moreover, our attempts to impute SV calls using tagging-SNPs captured 308 of 405 (76.0%) Venter bi-allelic SVs for which we could infer genotypes (Additional file 27) [19]. Based on population data, rare SVs with minimal allele frequency ≤ 0.05 showed the lowest correlation with surrounding SNPs, thus indicating that these SVs were least imputable (Figure 5). The fraction of imputable SVs will be even lower when multi-allelic and complex SVs are considered because the new mutation rate at these sites is higher.

## Discussion

Human geneticists have long sought to know the extent of genetic variation and here, in the most comprehensive analysis to date, we present the latest estimates of greater than 1% within an individual genome. Using multiple computational and experimental approaches, this study substantially expands on the SV map initially constructed by Levy and colleagues [1]; more than 80% of the total 48,777,466 structurally variable bases have not been reported from the original sequencing of the Venter genome.

Our study here differs from previous studies in many ways. Our mate-pair approach makes use of multiple different clone insert sizes, ranging from 2 to 37 kb, and this enables us to detect a wide size range of variants compared to previous paired-end mapping focused studies [15,17,18]. Furthermore, the long sequence reads used here increase alignment accuracy, and enable the identification of intra-alignment gaps. Using microarrays, we are able to identify large size variants that can be challenging to identify by sequencing.

Furthermore, our results highlight that each variation-discovery strategy has limitations and that no single approach can capture the entire spectrum of genetic variation, thus emphasizing the importance of applying multiple strategies in SV detection. Figure 4 shows that the variation distribution of other personal genome sequencing studies, which relied almost exclusively on NGS technology, is substantially lower than the Venter annotation across many size ranges.

There are still some regions, such as heterochromatin (Additional file 18) and highly identical segmental duplication regions, where all of the current approaches have limited detection capabilities. To prevent false discovery, we have used stringent alignment criteria, excluded alignments to multiple high-identity sequences, and will therefore likely miss variants within or flanking these sequences. Insufficient probe coverage and low intensity ratio fold-change also prevent microarrays from capturing CNV of highly repetitive sequences (for example, Alu elements). As such, we suspect there will be more variants to be discovered, but their ascertainment will require specialized experimental [18,28] and algorithmic [29-31] approaches. Further increases in read-depth can yield new variants. Indeed, the greatest relative number of SVs discovered in Venter is in the 10-kb size range (Figure 2), corresponding to the interval with the highest clone coverage [1] (Additional file 2). As expected, our results also show that using several libraries with different insert size leads to increased variation discovery.

The importance of SV to gene expression (direct and indirect) [32], protein structure [33], and chromosome stability [34,35] is being increasingly recognized in normal development and disease [9,20]. At the same time we show that SVs are: 1, grossly under-represented in published NGS sequencing projects; 2, not always imputable by SNP-based association; 3, ubiquitous along chromosomes impacting all known functional genomic features; and 4, often large, complex, and under negative or purifying selection [19,36]. Coupling these observations with conjectures that prophylactic decisions will be best informed by higher-penetrance rare alleles [10] and that common SNPs explain only a proportion of heritability [37] argue persuasively that SVs should gain more prominence in genomic medicine.

## Conclusions

Our results present the most thorough estimate to date of the total complement of genetic variation across the entire size spectrum in a human genome. Our findings indicate that, to date, NGS-based personal genome studies, despite having generated a significant amount of valuable genomic information, have captured only a fraction of SVs, with substantial gaps in discovery at specific points along the size range of variation. Our data indicate that SV discovery is largely dependent on the strategy used, and presently there is no single approach that can readily capture all types of variation and that a combination of strategies is required. The data also show that structural variation impact many genes that have been linked to human disease phenotypes, and that interpretation of these data is complex [38]. Current genotyping services offered in the personal genomics field do not always include screening for SVs, and we find that interpretation of current SNP-based screening may be significantly impacted by the existence of SVs. We also show that many SVs will not be amenable to capture using imputation strategies from high density SNP data, arguing for direct detection of SVs as a complement to SNP analysis.

## Materials and methods

### Sequencing-based analysis

The sequence data of J Craig Venter's genome (or the Venter genome) used for analysis was originally produced through experiments performed in the Venter *et al. *[39] and Levy *et al. *[1] studies. The sequence trace data and information files were downloaded from NCBI. In this study, we aligned 31,546,016 Venter sequences to the NCBI human genome assembly build 36 using BLAT [40]. For paired-end mapping, the optimal placement of clone ends was determined by a modified version of the scoring scheme used in Tuzun *et al. *[15]. We categorized mate-pairs that mapped less than three standard deviations from the expected clone size as putative insertions, greater than three standard deviations as putative deletions, and in the wrong orientation as putative inversions. We required each variant to be confirmed by at least two clones, and for indels, we required the clones to be from libraries of the same average insert size (2 kb, 10 kb or 37 kb). To identify small variants, the read alignment profiles were further examined for an intra-alignment gap with size greater than 10 bp. Two independent 'split-reads' were required to call a putative variant.

### Array-based analysis

An Agilent 24 million features CGH array set (Agilent 24 M) was designed with 23.5 million 60-mer oligonucleotide probes tiled along the NCBI build 36 assembly. The Venter genomic DNA was co-hybridized with the female sample NA15510 from the Polymorphism Discovery Resource [22]. The statistical algorithm ADM-2 by Agilent Technologies was used to identify CNVs based on the combined log _2 _ratios. Similar experimental procedures and analyses are described in other studies [7,41]. Additionally, a custom NimbleGen 42 million features CGH microarray (NimbleGen 42 M) was used in this study - its design, experimental procedures and data analysis have been described in detail elsewhere [19,22]. Venter genomic DNA was also co-hybridized with the sample NA15510. For both the Agilent 24 M and NimbleGen 42 M arrays, CNVs with >50% reciprocal overlap and opposite orientation of variants identified in NA15510 in Conrad *et al. *[19] were removed, as these were specific to the reference.

The Venter sample was also run on the Affymetrix SNP Array 6.0 and Illumina BeadChip 1 M genotyping arrays. We followed the protocol recommended by the manufacturers. For Affymetrix 6.0, the default parameters in the BirdSeed v2 algorithm were used to perform SNP calling. Partek Genomics Suite (Partek Inc., St. Louis, Missouri, USA), Genotyping Console (Affymetrix, Inc., Santa Clara, California, USA), BirdSuite [42] and iPattern (J Zhang *et al.*, manuscript submitted) were used to call CNVs. For Illumina 1 M, the SNP calling was done using the BeadStudio software. QuantiSNP [43] and iPattern were used to identify CNVs. For both platforms, only variants confirmed by at least two calling algorithms were included in the final set of calls.

The Agilent Custom Human 244 K CGH array (Agilent 244 K) was designed to target 9,018 sequences >500 bp in length that were annotated as 'unmatched' sequences in Khaja *et al. *[16]. CGH experiments were performed with genomic DNA from Venter and six HapMap samples, hybridized against reference NA10851. Feature extraction and normalization were performed using the Agilent feature extraction software. The programs ADM-1 in the DNA Analytics 4.0 suite (Agilent Technologies, Santa Clara, California, USA), and GADA [44] were independently used to call CNVs, and those that were confirmed by both algorithms were then used in this study.

### Non-redundant variant data set

To generate a non-redundant set of Venter variants, we combined the lists of SVs generated. For CNVs, to determine if two calls are the same, we required that they shared a minimum of 50% size reciprocal overlap; for inversions, we required that they shared at least one boundary. For those calls that were indicated to be the same variant, we recorded the one with the best size/boundary estimate (with preference given to assembly comparison, then split-read, NimbleGen-42 M, Agilent 24 M, mate-pair, Affymetrix 6.0, and Illumina 1 M, in that order). For this analysis, we excluded variants called in the custom Agilent 244 K arrays.

### PCR and quantitative real-time PCR validation

We used multiple computational and experimental approaches to validate SVs found in this project. PCR primers were designed to target flanking sequences of indels detected by sequencing-based methods, such that PCR products representing the different alleles can be differentiated on a 1.5% agarose gel. DNA from Venter and five HapMap individuals of European ancestry were tested in PCR experiments. Amplifications and deletions detected by CGH arrays were tested by qPCR. DNA from Venter and six additional control individuals were used to assess the variability in copy number. Each assay was run in triplicate and the *FOXP2 *gene was used as the reference for relative quantifications. See Additional file 12 for all primer sequences.

### FISH validation

To validate large variants, FISH experiments were performed using fosmid clones as probes on a lymphoblastoid cell line from Venter and seven other HapMap individuals. Five metaphases were first imaged to check for correct chromosome localization and hybridization, and then interphase FISH was performed to validate predicted inversions, similar to the protocol outlined in the Feuk *et al. *study [25] with the addition of the aqua probe, DEAC-5-dUTP (Perkin Elmer, Waltham, Massachusetts, USA; NEL455).

### Overlap analysis

Overlap with other datasets, genomic features and between subsets of data in the current paper was performed using custom PERL scripts. When comparing variants, two sites were considered overlapping if the reciprocal overlap among their estimated sizes was ≥ 50%. Data sources used for the annotations of overlaps with genomic features are listed in Additional file 17. To evaluate significance, we created 1,000 randomized sets of simulated variant calls and performed overlap analysis against the same data source. For each simulation, we recorded the number of instances where we observed a higher number of overlaps than the real variant data set. A *P*-value was computed as the fraction of simulations whose number of overlaps was greater than the number of real overlaps.

### Structural variation imputation

Using a cutoff of 50% reciprocal overlap, there were 405 sites of overlap between the Venter and genotyped, validated Genome Structural Variation (GSV) loci. The best *r*^2 ^value was computed between each of those GSV CNVs and a European's HapMap SNP in the neighboring genomic region. Here, we defined a minimum threshold of *r*^2 ^= 0.8, below which the Venter SVs were deemed not well imputed by SNP. Detailed description on genotyping, phasing, and tagging calls onto haplotypes defined by HapMap SNPs is presented in the Conrad *et al. *study [19].

### Data release

The sequence trace files generated from previous studies [1,39] can be obtained from the 'NCBI Trace Archive', using queries [CENTER_NAME = "JCVI" and SPECIES_CODE = "HOMO SAPIENS" and center_project = "GENOMIC-SEQUENCING-DIPLOID-HUMAN-REFERENCE-GENOME"], [INSERT_SIZE = 10201 and CENTER_NAME = "CRA" and SPECIES_CODE = "homo sapiens"], and [INSERT_SIZE = 1925 and CENTER_NAME = "CRA" and SPECIES_CODE = "homo sapiens"]. All of the microarray data generated in this study are available at the Gene Expression Omnibus (GEO) under the accession number [GEO:GSE20290]. The SV locations, size, and zygosity (when available), are reported in Additional files 3, 4, 5, 6, 7, 8 and 9, and a non-redundant set of variant data in the Venter genome is reported in Additional files 19, 20 and 21.

## Abbreviations

bp: base pair; CGH: comparative genomic hybridization; CNV: copy number variation; FISH: fluorescence *in situ *hybridization; GSV: Genome Structural Variation; indel: insertion/deletion; NCBI: National Center for Biotechnology Information; NGS: next generation sequencing; OMIM: Online Mendelian Inheritance in Man; qPCR: quantitative real-time PCR; SINE: short interspersed nuclear element; SNP: single nucleotide polymorphism; SV: structural variation.

## Authors' contributions

AWP, JRM, DP, DFC, HP, MEH, CL, JCV, EFK, SL, LF and SWS conceived and designed the experiments. AWP, JRM, JW, MAR, and LF performed the mate-pair and split-read analysis, as well as the Affymetrix 6.0 and Illumina 1 M experiments. HP and CL performed the Agilent 24 M experiments, while DP, DFC, and MEH did the NimbleGen 42 M experiments. All authors analyzed the data. AWP, LF and SWS wrote the paper. All authors read and approved the final manuscript.

## Supplementary Material

Additional file 1 **Genetic variation in sequenced genomes**. Click here for file

Additional file 2 **Clone library information**. Click here for file

Additional file 3 **Mate-pair variants and comparison with various data sets**. Click here for file

Additional file 4 **Split-read variants and comparison with various data sets**. Click here for file

Additional file 5 **Agilent 24 M variants and comparison with various data sets**. Click here for file

Additional file 6 **NimbleGen 42 M variants and comparison with various data sets**. Click here for file

Additional file 7 **Affymetrix 6.0 variants and comparison with various data sets**. Click here for file

Additional file 8 **Illumina 1 M variants and comparison with various data sets**. Click here for file

Additional file 9 **Custom Agilent 244 K copy number variants**. Click here for file

Additional file 10 **Custom Agilent 244 K copy number variable-scaffolds anchoring information**. Click here for file

Additional file 11 **Example of a PCR-validated insertion event with size 84 bp predicted by the split-read approach**. A pair of primers, separated by 497 bp was designed surrounding the insertion site. PCR was run with these primers, and the presence of the insertion was resolved by gel electrophoresis. Starting from the right, DNA from five European controls, DNA from Venter and a negative control were added in lanes 1 to 5, lane 6 and lane 7, respectively. Click here for file

Additional file 12 **List of validated variants and their primers and probes**. Click here for file

Additional file 13 **Example of a qPCR-validated gain in Venter relative to sample NA10851 as detected by the custom Agilent 244 K aCGH**. A 4.2-kb CNV was detected on the Celera scaffold GA_x5YUVVTY6, and by qPCR, we found that NA10851 had a heterozygous loss in that region, thus confirming a relative gain in Venter. Click here for file

Additional file 14 **A common inversion on 16p12.2 validated by FISH**. **(a) **A 2-Mb website schematic of the region. This 1.1-Mb inversion was detected by the mate-pair method in Venter as seen in track 'B_Clone'. The track 'Inversions' shows that this inversion was annotated in three other studies [15,17,18]. **(b) **An image of a four-color FISH experiment revealing that Venter is homozygous for the 16p12.2 inverted allele. Four differentially labeled fosmid probes were scored in >100 interphase FISH experiments and the order of the probes in Venter were found in the vast majority of experiments (including in seven HapMap controls from four different populations) to be in the yellow-green-blue-pink order. In the absence of the inversion, the order of the probes would be yellow-blue-green-pink as depicted in the assembly schematic. Therefore, as discussed in the main text our data suggest that the NCBI build 36 reference represents a rare allele, or may be incorrect. Click here for file

Additional file 15 **Comparative analysis of variants discovered in Levy *et al. ***[1]** and the current study**. The two graphs illustrate the proportion of SVs identified by the assembly comparison method, by our present combined multi-approach strategy (including mate-pair, split-read, CGH arrays and SNP arrays), and the proportion confirmed by both. The x-axis represents size range, while the numbers at the top indicate the total number of calls in a particular size range. As size increases, the number of variants called by assembly comparison decreases significantly, so this indicates that the method has limited sensitivity in detecting large calls. In contrast, our combined multi-approach strategy in the current study is more suitable in finding large variation. **(a) **Size distribution of gains. **(b) **Size distribution of losses. Click here for file

Additional file 16 **Cumulative distribution of probe coverage**. **(a) **Agilent 24 M array probe coverage across NimbleGen 24 M variants. The x-axis begins at 5 - the minimum requirement to call variants on the Agilent array. Hence, the majority of the unconfirmed NimbleGen variants (approximately 70%) were targeted less than five Agilent probes. **(b) **NimbleGen 42 M array probe coverage across Agilent 24 M variants. The x-axis begins at 10, which is the required number of probes for the NimbleGen array to make a call. Click here for file

Additional file 17 **A summary list of structural variants overlap with genomic features**. Click here for file

Additional file 18 **Genome-wide distribution of large SVs in Venter**. The sites of 2,772 SVs whose position spans >1 kb are shown. Red bars represent insertion or duplication, blue bars represent deletions, and green bars represent inversions. Click here for file

Additional file 19 **A non-redundant set of Venter insertions and duplications**. Click here for file

Additional file 20 **A non-redundant set of Venter deletions**. Click here for file

Additional file 21 **A non-redundant set of Venter inversions**. Click here for file

Additional file 22 **List of Venter gains that overlap with exons of RefSeq genes**. Click here for file

Additional file 23 **List of Venter losses that overlap with exons of RefSeq genes**. Click here for file

Additional file 24 **List of Venter gains that overlap with exons of OMIM genes**. Click here for file

Additional file 25 **List of Venter losses that overlap with exons of OMIM genes**. Click here for file

Additional file 26 **A detailed list of genes that are completely encompassed with non-redundant gains and losses**. Click here for file

Additional file 27 **Comparison of Venter SVs with population-based genotyped and SNP-imputable CNVs**. Click here for file
