# Supplementary figures and images for: Towards a comprehensive structural variation map of an individual human genome
Source: Genome Biol. 2010 May 19;11(5):R52. doi: 10.1186/gb-2010-11-5-r52 (PMC2898065; doi:10.1186/gb-2010-11-5-r52)

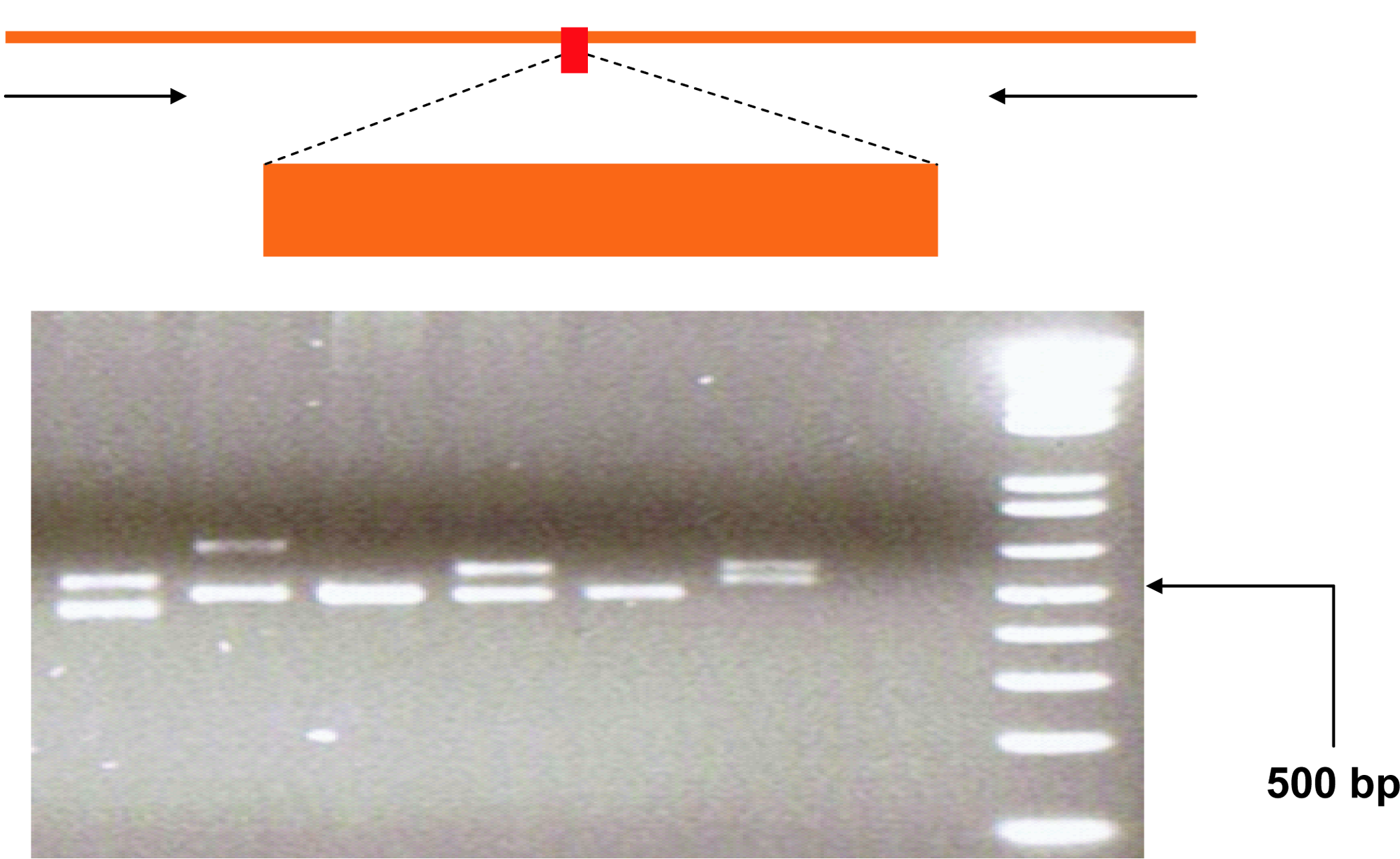

Supplement: Additional file 11 — Example of a PCR-validated insertion event with size 84 bp predicted by the split-read approach. A pair of primers, separated by 497 bp was designed surrounding the insertion site. PCR was run with these primers, and the presence of the insertion was resolved by gel electrophoresis. Starting from the right, DNA from five European controls, DNA from Venter and a negative control were added in lanes 1 to 5, lane 6 and lane 7, respectively. [file gb-2010-11-5-r52-S11.TIFF]

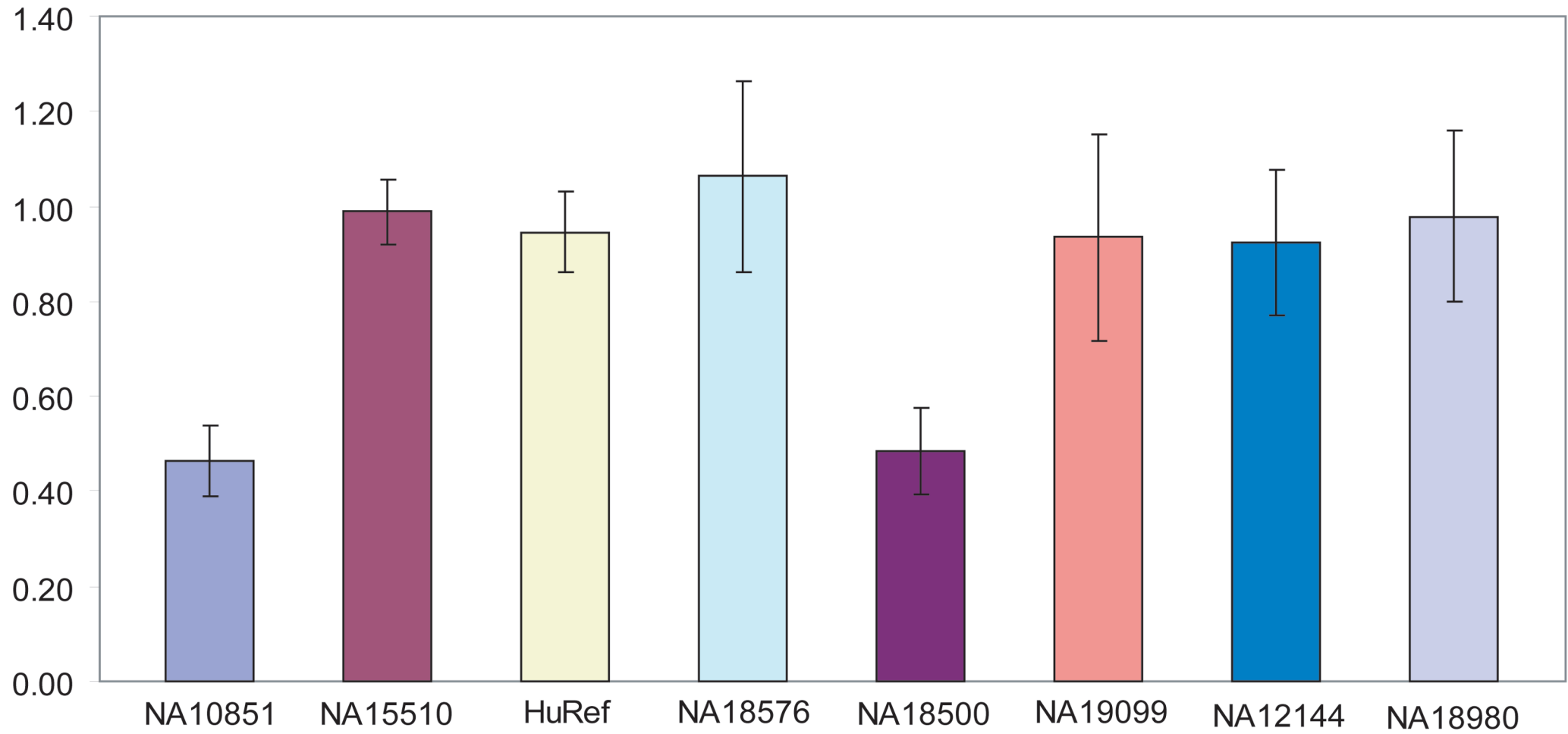

Supplement: Additional file 13 — Example of a qPCR-validated gain in Venter relative to sample NA10851 as detected by the custom Agilent 244 K aCGH. A 4.2-kb CNV was detected on the Celera scaffold GA_x5YUVVTY6, and by qPCR, we found that NA10851 had a heterozygous loss in that region, thus confirming a relative gain in Venter. [file gb-2010-11-5-r52-S13.PDF]

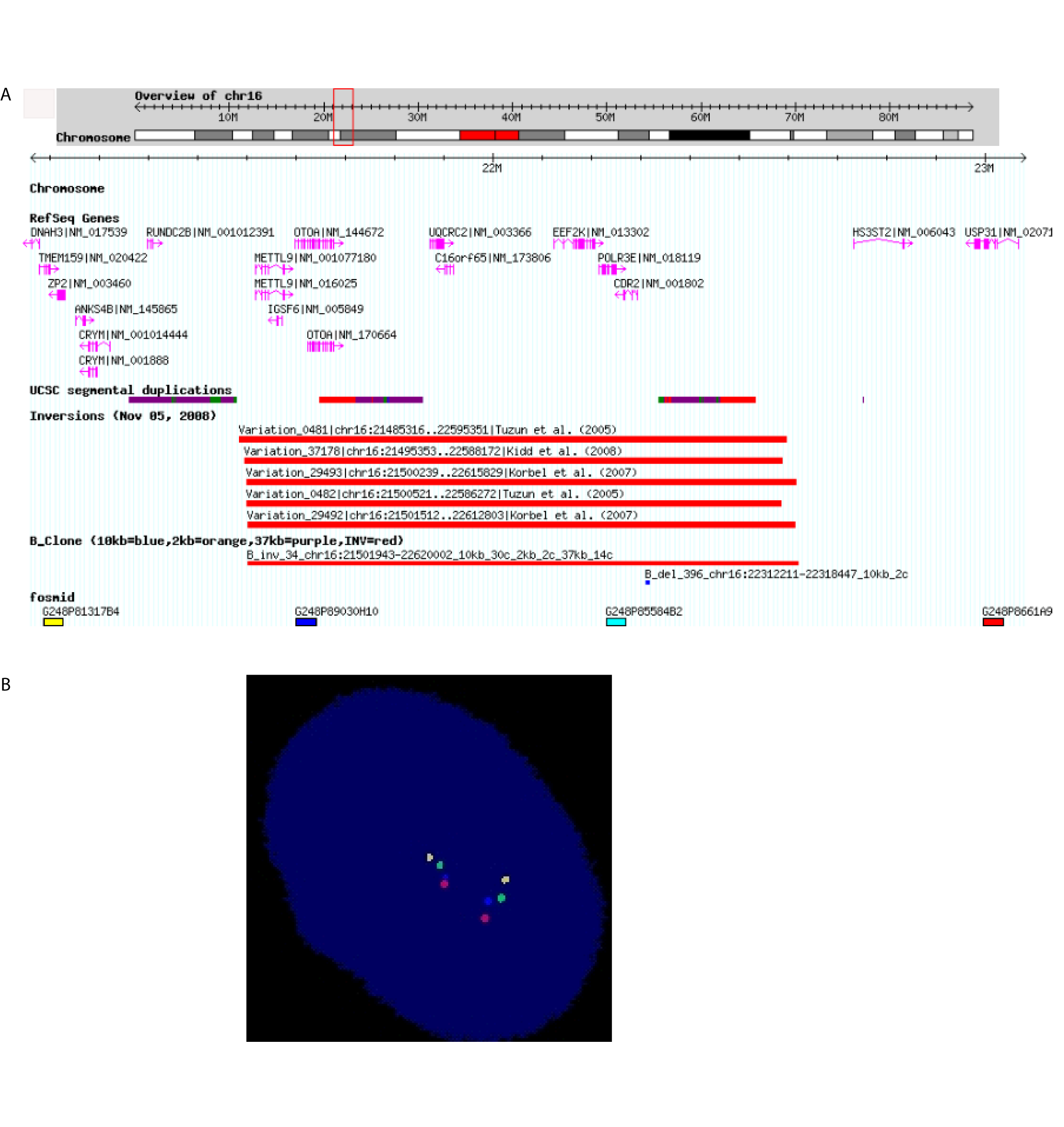

Supplement: Additional file 14 — A common inversion on 16p12.2 validated by FISH. (a) A 2-Mb website schematic of the region. This 1.1-Mb inversion was detected by the mate-pair method in Venter as seen in track 'B_Clone'. The track 'Inversions' shows that this inversion was annotated in three other studies [15,17,18]. (b) An image of a four-color FISH experiment revealing that Venter is homozygous for the 16p12.2 inverted allele. Four differentially labeled fosmid probes were scored in >100 interphase FISH experiments and the order of the probes in Venter were found in the vast majority of experiments (including in seven HapMap controls from four different populations) to be in the yellow-green-blue-pink order. In the absence of the inversion, the order of the probes would be yellow-blue-green-pink as depicted in the assembly schematic. Therefore, as discussed in the main text our data suggest that the NCBI build 36 reference represents a rare allele, or may be incorrect. [file gb-2010-11-5-r52-S14.TIFF]

A

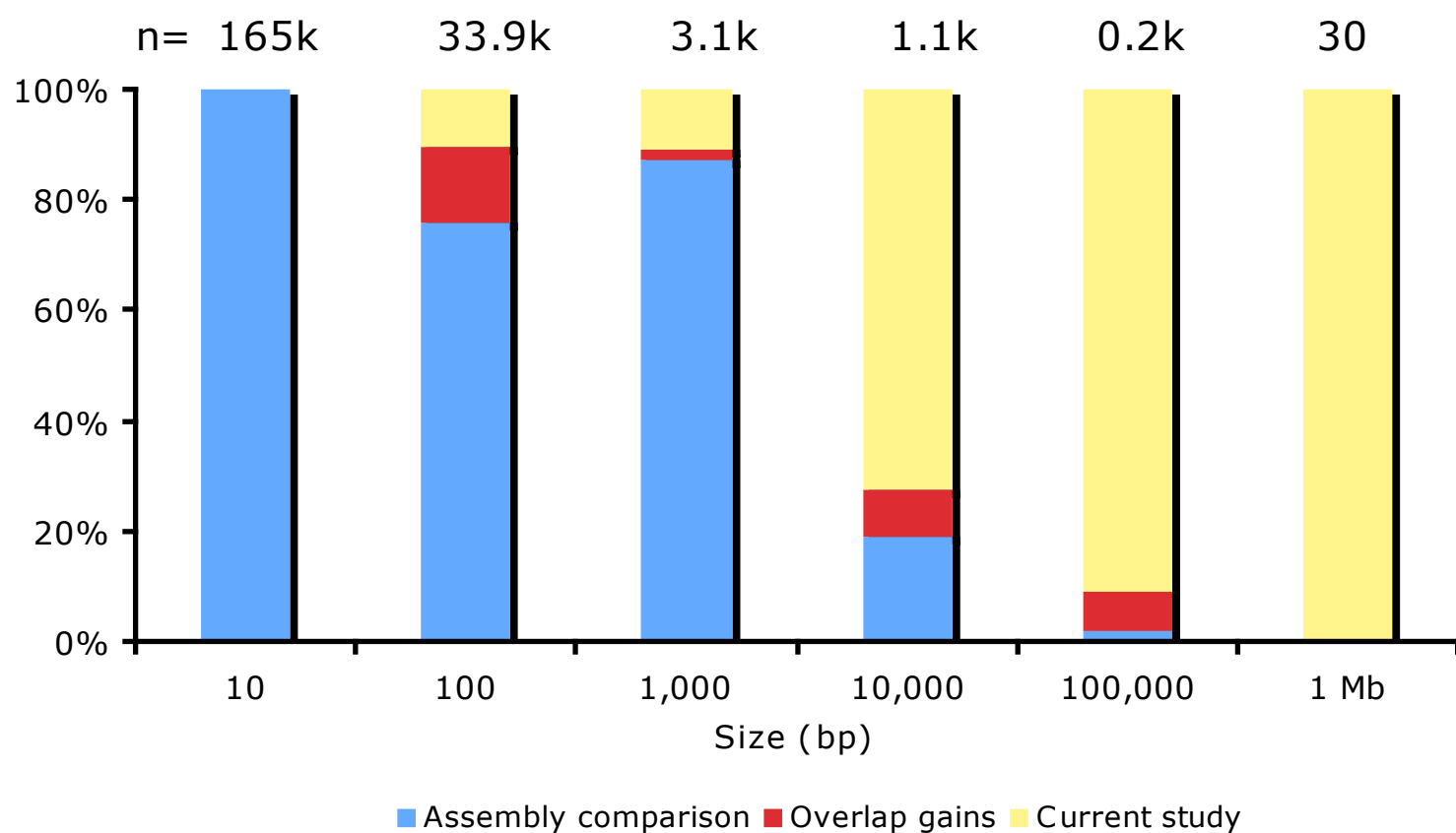

B

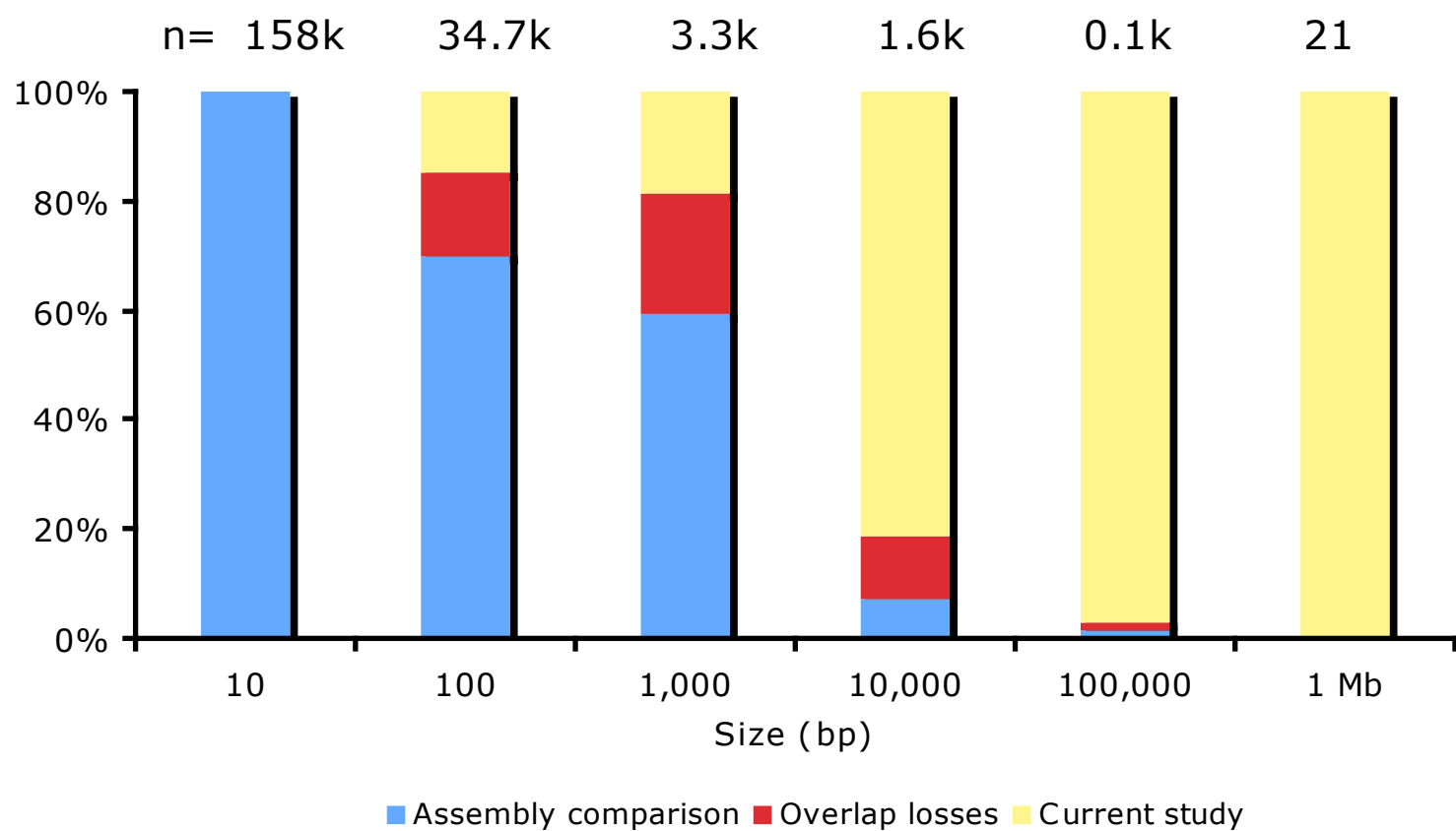

Supplement: Additional file 15 — Comparative analysis of variants discovered in Levy et al. [1] and the current study. The two graphs illustrate the proportion of SVs identified by the assembly comparison method, by our present combined multi-approach strategy (including mate-pair, split-read, CGH arrays and SNP arrays), and the proportion confirmed by both. The x-axis represents size range, while the numbers at the top indicate the total number of calls in a particular size range. As size increases, the number of variants called by assembly comparison decreases significantly, so this indicates that the method has limited sensitivity in detecting large calls. In contrast, our combined multi-approach strategy in the current study is more suitable in finding large variation. (a) Size distribution of gains. (b) Size distribution of losses. [file gb-2010-11-5-r52-S15.PDF]

A

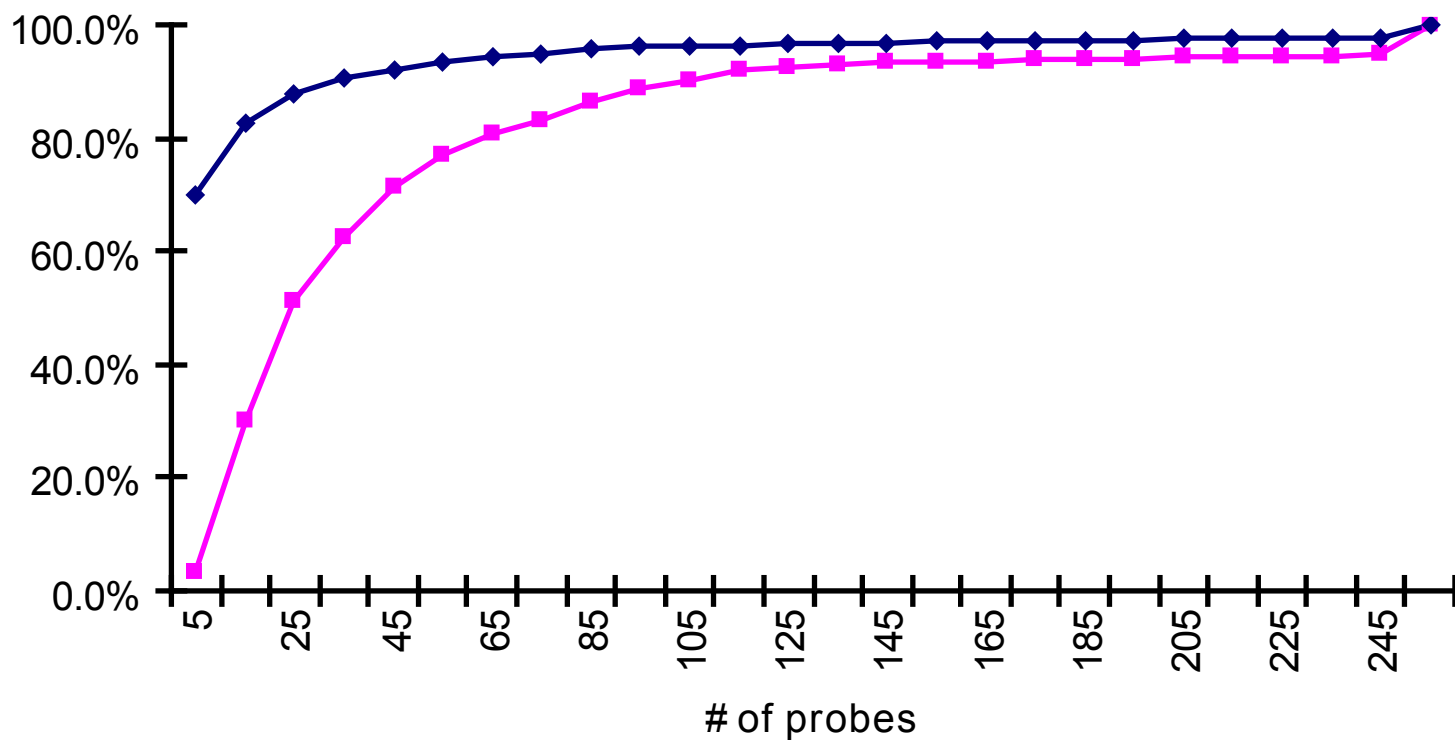

Confirmed cum % Unconfirmed cum %

B

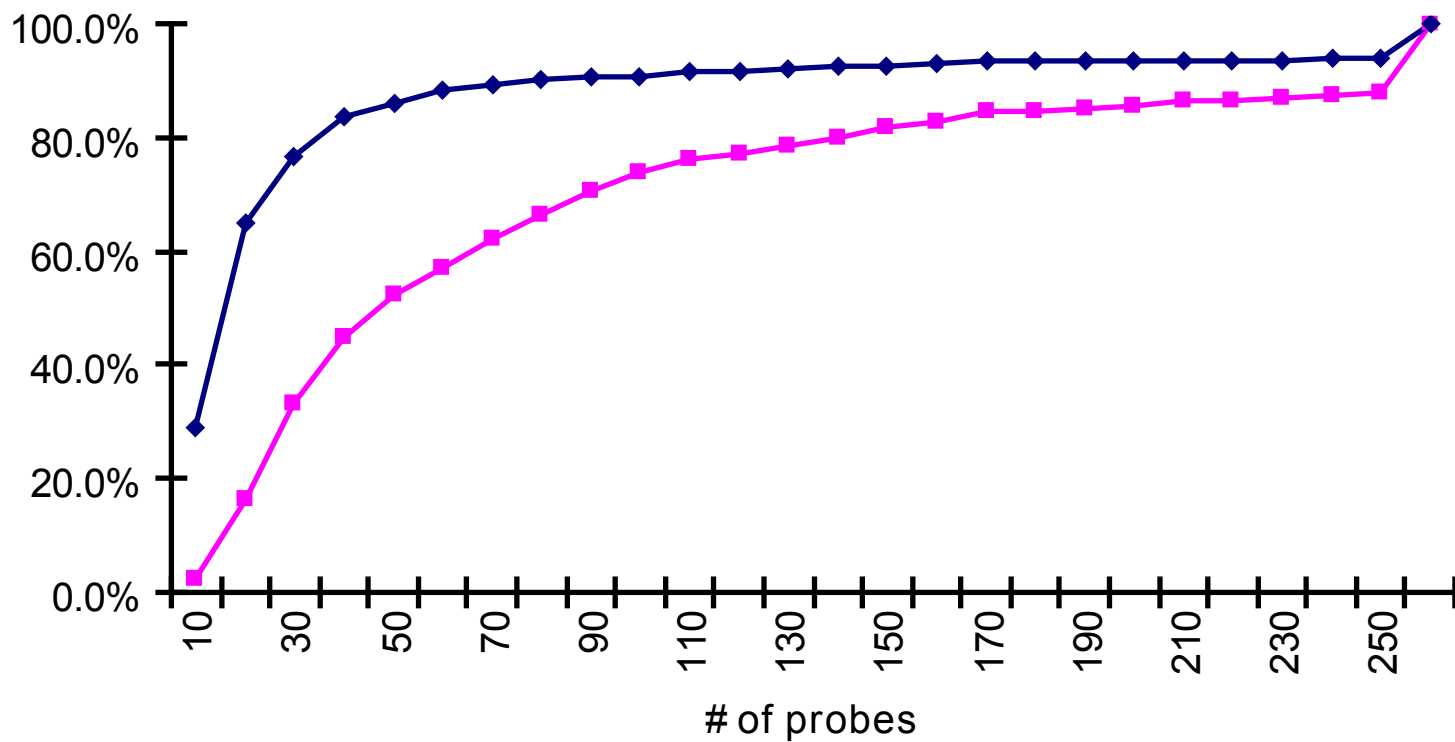

Confirmed cum % Unconfirmed cum %

Supplement: Additional file 16 — Cumulative distribution of probe coverage. (a) Agilent 24 M array probe coverage across NimbleGen 24 M variants. The x-axis begins at 5 - the minimum requirement to call variants on the Agilent array. Hence, the majority of the unconfirmed NimbleGen variants (approximately 70%) were targeted less than five Agilent probes. (b) NimbleGen 42 M array probe coverage across Agilent 24 M variants. The x-axis begins at 10, which is the required number of probes for the NimbleGen array to make a call. [file gb-2010-11-5-r52-S16.PDF]

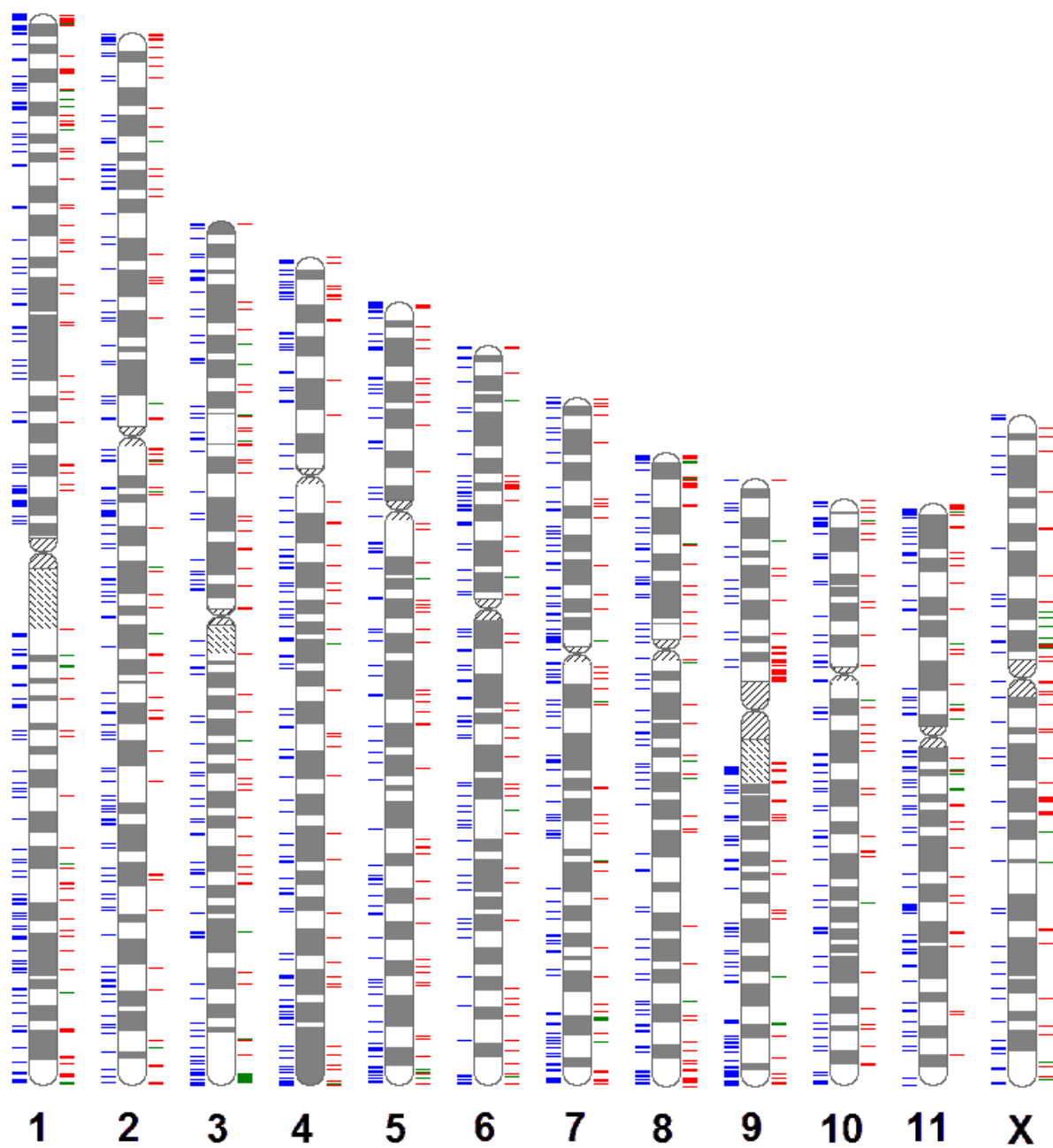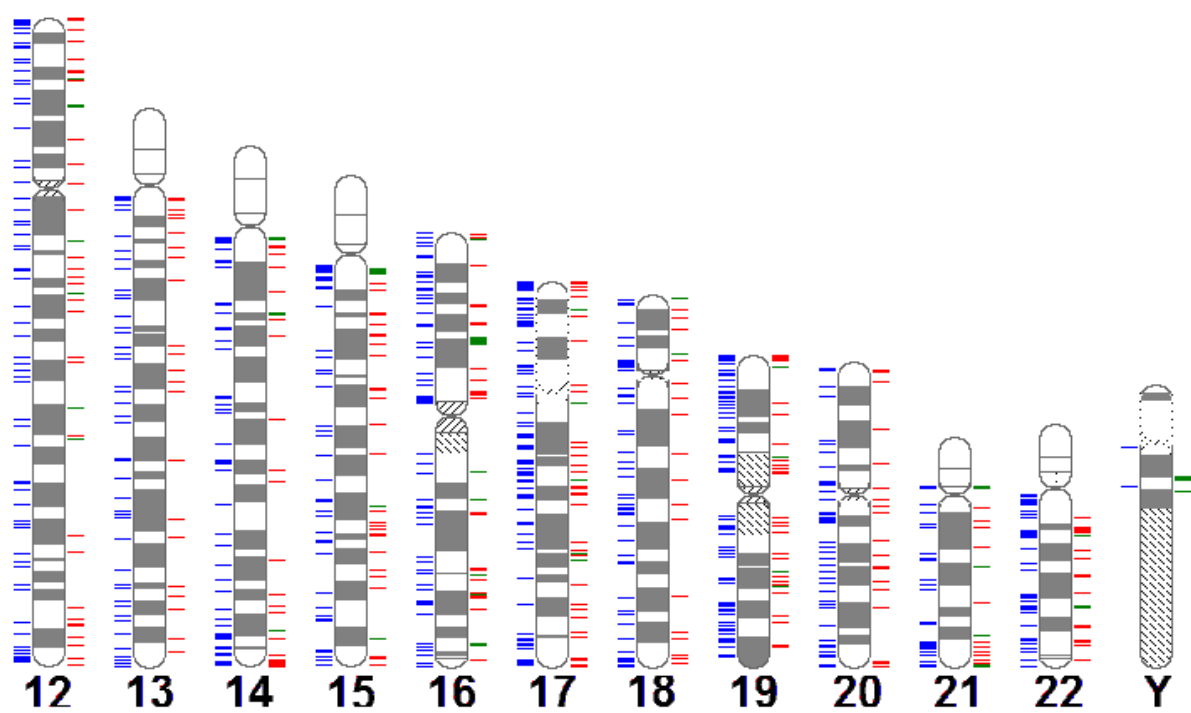

Supplement: Additional file 18 — Genome-wide distribution of large SVs in Venter. The sites of 2,772 SVs whose position spans >1 kb are shown. Red bars represent insertion or duplication, blue bars represent deletions, and green bars represent inversions. [file gb-2010-11-5-r52-S18.PDF]
